# Optical Aberrations of Guinea Pig Eyes

**DOI:** 10.1167/iovs.61.10.39

**Published:** 2020-08-21

**Authors:** Sarah Elizabeth Singh, Christine Frances Wildsoet, Austin John Roorda

**Affiliations:** 1 School of Optometry and Vision Science Graduate Program, University of California, Berkeley, Berkeley, California, United States

**Keywords:** myopia, refractive error, optics

## Abstract

**Purpose:**

The guinea pig is widely used in studies of refractive error development and myopia which often involve experimental optical manipulations. The study described here investigated the optical quality of the guinea pig eye, for which there are limited data, despite its fundamental importance to understanding visually guided eye growth.

**Methods:**

The ocular aberrations of eight adolescent New Zealand pigmented guinea pigs (6–11 weeks old) were measured after cycloplegia using a custom-built Shack–Hartmann aberrometer and fit with a Zernike polynomial function to the 10th order (65 terms). The optical quality of their eyes was assessed in terms of individual Zernike coefficients, and data were further analyzed to derive root-mean-square (RMS) wavefront errors, modulation transfer functions (MTFs), point spread functions (PSFs), Strehl ratios, and depth of focus. A 4-mm pupil was used in all computations. The derived data are compared with equivalent data from normal young adult human eyes.

**Results:**

The guinea pigs exhibited low hyperopia and a small amount of positive spherical aberration, with other aberration terms decreasing with increasing order. Their average depth of focus, estimated from through-focus modulation, was 3.75 diopters. The RMS wavefront error of the guinea pig eye was found to be larger than that of the human eye for the same pupil size, reflecting a higher degree of aberrations, although the PSF (area) on the retina was smaller and sharper due to its shorter focal length. The radial average best-focus MTF derived for the guinea pig eye showed good performance at very low spatial frequencies, with a steeper decline with increasing frequency than for the human eye, dropping below 0.3 at 9 cpd. When converted to linear units (cycles/mm), the guinea pig eye had a higher spatial frequency cutoff and a slight contrast advantage for low spatial frequencies compared to the human eye.

**Conclusions:**

The optical quality of the guinea pig eye is far superior to their reported behavioral visual acuity. This implies a neuroanatomical limit to their vision, which contrasts with the close match of optical and neural limits to spatial resolution in human eyes. The significance for eye growth regulation of the relative optical advantages exhibited by guinea pig eyes, when optical quality is expressed in linear rather than angular retinal units, warrants further consideration.

The guinea pig has emerged as an important mammalian model for studies of refractive error development and myopia. As is typical of early ocular development in most animals, young guinea pigs undergo emmetropization,^1^^,^^2^ and this process appears to be visually guided.^3^^–^^6^ For example, young guinea pigs respond to defocus-induced blur with compensatory adjustments to eye growth.^3^ Further evidence that their visual system can detect and respond to imposed defocus comes from the observation that young guinea pigs are able to accommodate, implying that the guinea pig has a visually (retina)-guided focusing mechanism.^7^

As a model for studying visually guided eye growth regulation, knowledge of the retinal image quality of the developing guinea pig eye is important. Rodents are typically nocturnal with small eyes and relatively poor vision compared to other mammals, relying instead on highly developed senses of olfaction and hearing.^8^ However, the guinea pig is one of a small number of exceptions, being a crepuscular rodent that is most active at dawn and dusk; it also has relatively large eyes compared to mice and rats. Although this difference in eye length offers the potential for greater spatial resolving power, the visual acuity of the guinea pig, based on behavioral measures, is reported to be relatively poor, between 1.0 cycles per degree (cpd) (Ostrin LA, et al. *IOVS* 2011;52:ARVO E-Abstract 6296) and 2.7 cpd,^9^ making it only slightly better than that of mice (0.5 cpd)^10^ and much lower than that of chicks (6–8.6 cpd)^11^ and humans (30–60 cpd).^12^ Interestingly, albino guinea pigs and pigmented guinea pigs have very similar visual spatial resolution thresholds, despite the increased light scatter in albino eyes (Ostrin LA, et al. *IOVS* 2011;52:ARVO E-Abstract 6296), raising the possibility that the optical quality of the guinea pig eye is inherently poor. Characterization of the high-order aberrations of the guinea pig eye can help to model image transfer in the guinea pig eye and inform the limits of its spatial resolution, with important implications for studies involving experimental visual manipulations.

Animal models of myopia assume an ability of ocular growth regulatory mechanisms to respond to altered visual experience, including the effects of imposed defocus. The ability of the retina to detect such changes is determined in part by the nature and magnitude of naturally occurring optical aberrations, which in turn determine retinal image quality and the depth of focus of the eye. Therefore, the effects of focusing errors on eye growth will be very different for an eye that is diffraction limited compared to one that is highly aberrated. At this time, relevant studies involving the guinea pig are limited to just one paper,^13^ which used quantitative three-dimensional spectral optical coherence tomography (OCT) and laser ray tracing (LRT) to quantify the ocular optical aberrations within a central 2-mm pupil zone of four pigmented, adolescent animals (ages 30–40 days).

The study reported here made use of a Shack–Hartmann aberrometer, which allows for rapid, accurate, and objective measurements of wave aberrations. Wave aberration data collected from one eye of each of eight young guinea pigs were used to derive image quality metrics over a 4-mm pupil that were compared with known wavefront error trends in humans.

## Methods

A total of nine pigmented guinea pigs (*Cavia porcellus*) were used in this study. One of these guinea pigs was a cooperative 2-year old subject, which was used to test and refine the measurement protocol for the study. Eight additional adolescent guinea pigs (6–11 weeks of age; three sets of siblings) were used in the main study. Guinea pigs were housed in standard guinea pig cages under a 12-hour light/dark cycle in animal facilities of the University of California, Berkeley. All animal care and treatments conformed to the ARVO Statement for the Use of Animals in Ophthalmic and Vision Research. Experimental protocols were approved by the Animal Care and Use Committee at the University of California, Berkeley.

Ocular aberrations were measured with a custom-built Shack–Hartmann aberrometer, a widely accepted method for measuring monochromatic high-order aberrations of the eye.^14^ The aberrometer used an 840-nm light source as the laser beacon, with a power of about 10 µW. A 7.6-mm focal length lenslet array sampled the pupil in a rectilinear grid with 0.375-mm spacing, offering ∼90 sampled points across a 4-mm pupil. Custom software was used for image capture, image analysis, and computing the weights of the Zernike polynomial coefficients used to describe the wavefront.^15^ Wavefront aberrations were fit with an OSA Standard Zernike polynomial function to the 10th order (65 terms).^16^

**Figure 1.**
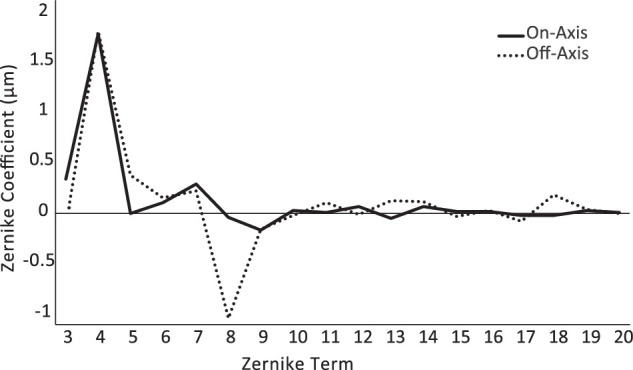
Mean Zernike coefficients for terms 3 to 20 for one representative guinea pig (2-year-old male), measured on-axis (*solid line*) and off-axis (*dashed line*). Profiles were similar except for a higher level of coma (eighth term) in the latter case. This guinea pig was excluded from further analysis due to his older age compared to the other animals. Calculations used a 4-mm pupil and 550-nm wavelength.

Measurements were limited to the left eyes of the guinea pigs, which were cyclopleged with topical 1% cyclopentolate, instilled 30 minutes prior to imaging, and were otherwise untreated. Pupil sizes ranged from 4.10 to 5.56 mm across animals after cycloplegia. The guinea pigs were handheld but not anesthetized for image capture. To correct for the superiorly tilted optical axis of the guinea pig eye, animals were held at a slight angle in compensation to ensure measurements were taken along an axis perpendicular to their pupil plane. The lack of excessive of coma ($Z_{3}^{1}$, $Z_{3}^{-1}$), as seen in Figure 1, was used as an indicator of valid (on-axis) alignment during measurement in accepting images for use in further analyses. Five to 10 images were collected per eye.

For each guinea pig subject in the study, digital images (uncompressed TIFF format) of the spot patterns were collected for use in analyses. For each image, a series of files were created to include the Zernike coefficients for a range of pupil sizes from 1.5 mm to the maximum pupil size, in 0.5-mm increments.

All analyses were performed over a 4-mm pupil to avoid the potential confounding effects of inter-animal variation in pupil size and to allow for direct comparison of the optical properties of all eyes. Note that the raw images occasionally exhibited an elongated or dual spot pattern, consistent with reflections from both the inner retinal surface and a deeper retinal layer (presumed photoreceptors).^17^ In these cases, care was taken during image analysis to choose spots originating from the deeper layer. Reported data represent averages derived from at least five individual measurements (images).

Zernike coefficients for the wave aberrations from 18 de-identified adult human subjects (mean age, 26.4 ± 4.3 years; range, 22–40 years) were selected from a previously published dataset and reanalyzed to compare with the guinea pig data.^15^ These data represent a subset of data from a much larger dataset representing 74 human eyes, with the selected data uniformly distributed across the complete dataset, avoiding the extremes (highest and lowest root-mean-square [RMS] values). Each set of data represents the average of the Zernike coefficients from three high-quality images.

All wavefront analyses were performed using custom-written software in MATLAB (MathWorks, Natick, MA, USA). As per the OSA Standard Zernike polynomial, terms 3 to 5 are considered second-order aberrations and account for defocus and astigmatism, which are typically the largest ocular aberrations. Terms 6 to 9 (trefoil and coma), 10 to 14 (including spherical aberration), and 15 to 20 comprise the third, fourth, and fifth orders, respectively. The optical quality of the eyes was assessed in terms of these individual Zernike coefficients and further analyzed in terms of RMS wavefront errors for these different orders.^16^ Point-spread functions (PSFs) and modulation transfer functions (MTFs) were also computed from the derived Zernike polynomials using a wavelength of 550 nm to generate metrics of image quality. Although the wavefronts were measured using a wavelength of 840 nm, the effects of chromatic dispersion and measurement wavelength are largely confined to the defocus term, with high-order aberrations changing very little as a function of wavelength.^18^^–^^22^ Therefore, no specific correction for the measurement wavelength was made in analyses of image quality, after adjustment of the defocus (refractive error) term. The refractive error for the 840-nm wavelength was estimated to be approximately 4.20 diopters (D) more hyperopic than that for the 550-nm wavelength, using a reduced eye model in combination with the method described by Hughes^23^ and schematic guinea pig eye model parameters from Howlett and McFadden.^1^

The PSFs were used to assess image quality by convolving the image with the letter E for a qualitative assessment and by generating Strehl ratios for a quantitative assessment. The Strehl ratio is defined as the ratio of the peak aberrated image intensity of a point source compared to the maximum attainable intensity of a diffraction-limited system for the same pupil size. A higher Strehl ratio corresponds to better image representation. The depth of focus of the guinea pig eye was calculated from the corresponding Strehl ratio by computationally adjusting the defocus term in 0.25-D steps from –5 to +5 D, where the 0-D defocus condition represents each subject's peak Strehl ratio. The depth of focus was computed as the width of the through-focus Strehl ratio at half of its maximum height. The PSFs were also used to generate the ocular modulation transfer function, which represents the optical contribution to the contrast sensitivity function, reflecting the extent to which details from objects are captured in the retinal image.

## Results

**Figure 2.**
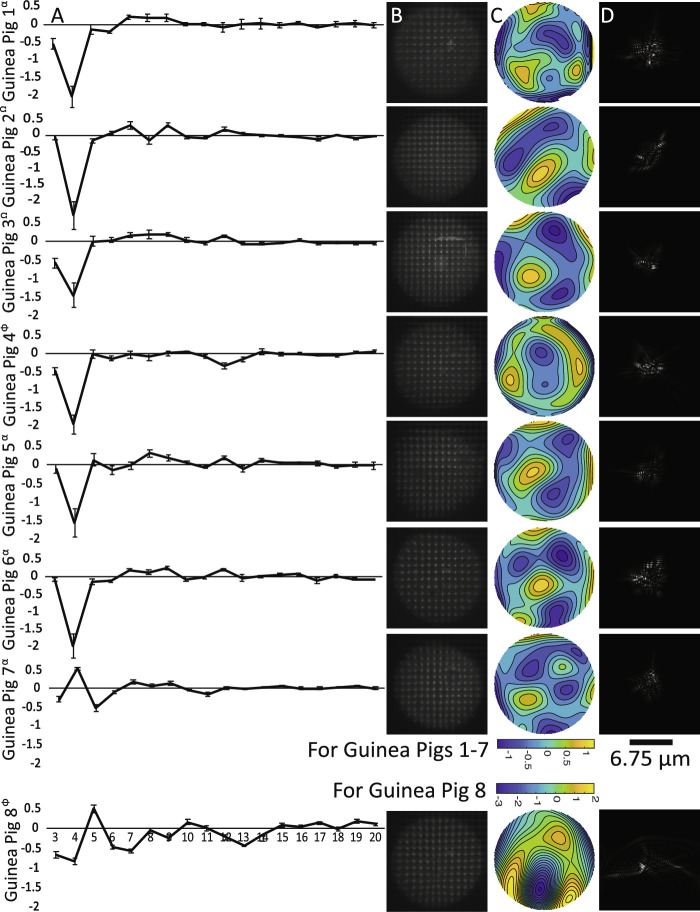
Column A shows mean Zernike coefficients for terms 3 to 20, derived from at least five images, for each of eight adolescent guinea pigs. Littermates are indicated with *superscript symbols*. *Error bars* represent standard deviations. Column B shows the raw Shack–Hartmann wavefront sensor spot patterns, column C shows the derived wavefront aberration patterns (color scale in mm), and column D shows the point spread functions for each guinea pig measured. Calculations used a 4-mm pupil and 550-nm wavelength.

Representative Shack–Hartmann images, along with derived wavefront aberration maps, point-spread functions, and mean Zernike coefficients are shown for each guinea pig subject in Figure 2. Only second- through fifth-order terms (coefficients 3–20) are shown. One of the guinea pig subjects (#8) showed significantly increased aberrations relative the other subjects, and, although its data are included in Table 1 and Figure 2, the data were otherwise excluded from further analyses. Its increased aberrations were subsequently discovered to be due to a previously undetected cataract.

**Table 1.** High-Order RMS Wavefront Errors and Maximum Strehl Ratios for Adolescent Guinea Pigs

	Guinea Pig							
	1	2	3	4	5	6	7	8
RMS error	0.56	0.55	0.39	0.45	0.49	0.50	0.36	1.06
Strehl ratio	0.019 (+0.25 D)	0.027 (–0.25 D)	0.031 (0 D)	0.028 (–1.50 D)	0.026 (–0.50 D)	0.025 (–0.75 D)	0.027(0 D)	0.014 (–0.25 D)

The defocus level corresponding to the peak Strehl ratio is noted in parentheses.

Overall, the guinea pigs exhibited only small refractive errors. Spherical equivalent refractive errors (computed from the second-order defocus terms for a wavelength of 550 nm), ranged from –0.84 to +4.23 D, with a mean refractive error of +2.54 ± 1.6 D (mean ± SD). As a rule, the defocus state that gave the minimum RMS errors (optimal image quality based on all Zernike coefficients) was zero for all guinea pigs, as shown in Table 1. However, the maximum Strehl ratio (optimal image quality based on PSF) did not necessarily match the defocus state with the minimum RMS error. The defocus state with the maximum Strehl ratio had non-zero values for all but animals #3 and #7. Among the seven guinea pigs, the minimum RMS wavefront errors ranged from 0.047 to 0.072, and the corresponding peak Strehl ratios ranged from 0.019 to 0.031. Nonetheless, there is generally good agreement for both of these image quality metrics, even though the RMS error is influenced by all Zernike coefficients, without weighing the relative importance of their impact on vision, leading to some differences in judgment of the optimal image quality.^24^

**Figure 3.**
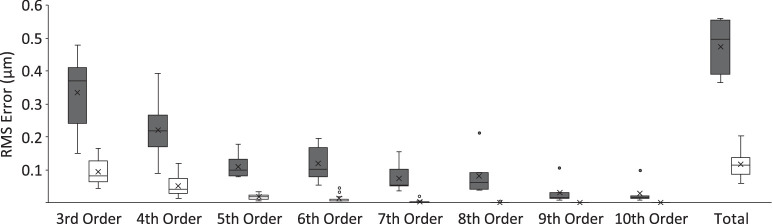
RMSs plotted as a function of wavefront order representing the left eyes of seven adolescent guinea pigs (*gray*) and 18 young adult humans (*white*). Guinea pig #8 was excluded from this analysis because he was found to have previously undetected cataracts and had the largest RMS error, 1.06, which was far outside the range of any of the other animals tested. Calculations used a 4-mm pupil and 550-nm wavelength.

The RMS wavefront errors as a function of order are plotted in Figure 3, with equivalent data from human eyes included for comparison. For both guinea pig and human eyes, the high-order RMS errors decrease with increasing order; however, RMS values were consistently larger for the guinea pig eye by approximately four times (0.47 vs. 0.11 for the guinea pigs and humans, respectively). Among the guinea pigs, guinea pig #8, which was found to have cataracts, had the largest RMS error and smallest peak Strehl ratio, 1.06 and 0.014, respectively, consistent with poor optical and image quality.

**Figure 4.**
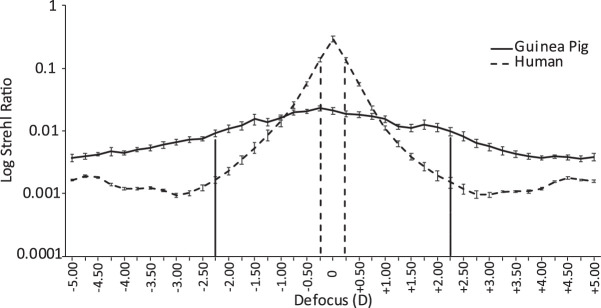
Through-focus estimation of depth of focus (DOF) derived from Strehl ratios centered around their respective peaks. The DOF estimated for seven guinea pig eyes was 4.50 D (–2.25 to +2.25 D), much larger than the estimated DOF for 18 human eyes, 0.50 D (–0.25 to +0.25 D). *Error bars* represent the standard errors of the mean. Calculations used a 4-mm pupil and 550-nm wavelength.

Guinea pig and human eyes were also compared in terms of their ocular depth of focus. Strehl ratio data for a 4-mm pupil size are shown plotted against defocus in Figure 4 for both guinea pig and human eyes. These graphical analyses were also used to estimate the ocular depth of focus using the through-focus technique (full-width at half-maximum Strehl ratio). Consistent with the comparatively large RMS errors across all orders for the guinea pig eye, the average depth of focus of the guinea pig eye was also larger, by approximately four times (3.75 vs. 0.50 D), compared to the human eye.

**Figure 5.**
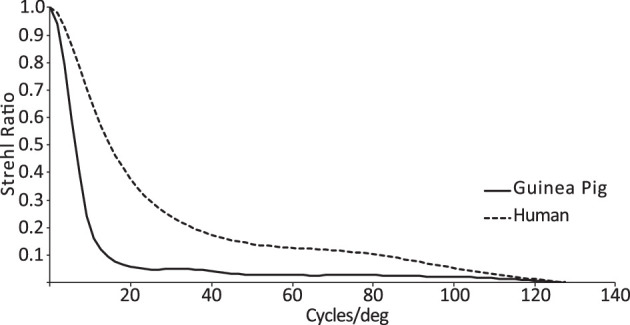
The radial average MTFs for guinea pig and human eyes. Human eyes are superior to guinea pig eyes in preserving contrast across most spatial frequencies. Calculations used a 4-mm pupil and 550-nm wavelength.

The radial average best-focus MTFs for both guinea pig and human eyes are shown in Figure 5, corrected for best focus (maximum Strehl ratio) over a 4-mm pupil. Both human and guinea pig eyes performed well at very low spatial frequencies; however, the guinea pig eye showed a much steeper decline with increasing spatial frequency, with contrast dropping below 0.3 at 9 cpd, whereas this point was not reached until 25 cpd for the human eye.

The area under an MTF curve represents a way of characterizing the modulation properties of an imaging system, capturing both spatial contrast and resolution information. These data for representative guinea pig and human eyes are summarized in Table 2. In both cases, the area under the MTF is largest for the smallest pupil size, decreasing thereafter. These results suggest that the smallest pupil size offers the best image quality overall, even though the cutoff frequency decreases in parallel with decreasing pupil size for the guinea pig eye.

**Table 2.** Calculated Area Under the Radial Average MTF Curve Corresponding to the Maximum Strehl Ratio Value for Representative Young-Adult Human and Adolescent Guinea Pig Eyes

	Pupil Size (mm)				
	2	3	4	5	6
Human	20.58	17.95	17.68	15.85	12.84
Guinea pig	7.94	6.14	6.62	n/a	n/a

**Figure 6.**
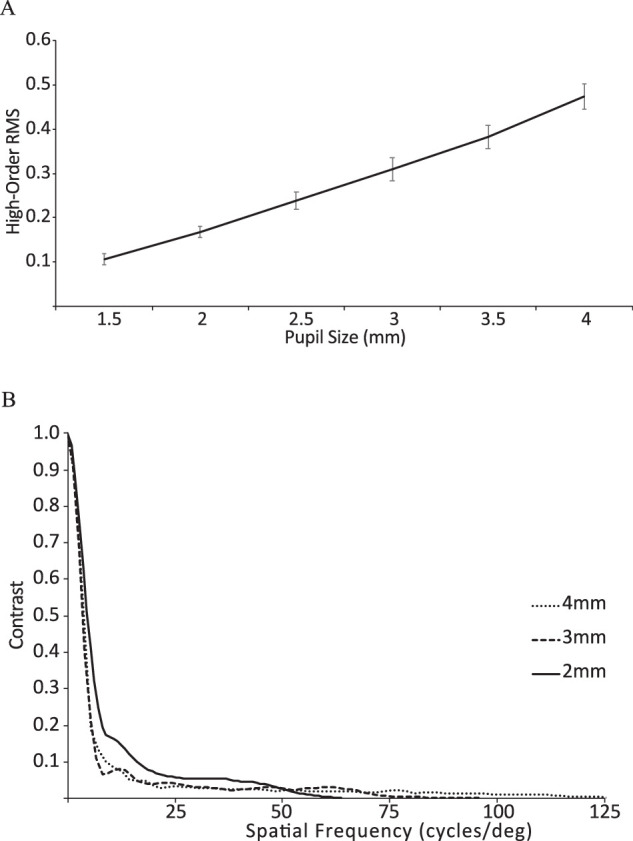
(**A**) Mean RMS wavefront error plotted as a function of pupil size for guinea pig eyes (*n* = 7). RMS error increased with increasing pupil size. *Error bars* represent standard errors of the mean. (**B**) Radial average MTFs for a representative guinea pig eye (guinea pig 6) and 2- to 4-mm pupil sizes. With decreasing pupil size, contrast was better preserved, up to 50 cpd. Calculations used a 550-nm wavelength.

The influence of pupil size on both RMS errors at best focus and the radial average best-focus MTF of the guinea pig eye is illustrated graphically in Figures 6A and 6B, respectively. Data from one representative guinea pig (#6) were used to generate the latter plot (Fig. 6B). For pupils ranging in size from 1.5 to 4 mm in diameter, the mean RMS error increased rapidly with increasing pupil size, from a mean value of 0.107 µm for a 1.5-mm pupil size to 0.475 µm for a 4-mm pupil size (Fig. 6A). Although the ability of a diffraction-limited optical system to transfer contrast of an object to an image improves with increasing pupil size, the opposite is true for the guinea pig eye, which represents a significantly aberrated optical system. Specifically, eyes with small pupils perform best, with contrast declining more rapidly with increasing spatial frequency for larger pupils. On the other hand, eyes with smaller pupils appear less sensitive to higher spatial frequencies; as indicated above, the high spatial frequency cutoff decreased proportionally with decreasing pupil size.

## Discussion

The study reported here made use of a Shack–Hartmann aberrometer to optically profile the eyes of young adolescent guinea pigs. The power of this approach is its ability to capture all optical aberrations, including second-order Zernike terms, which are the limit of information collected with traditional refractometry methods. The adolescent guinea pigs in this study proved to be slightly hyperopic (+2.54 ± 1.6 D), as reported in previous studies for similarly aged animals using retinoscopy.^1^^,^^2^ Although the observation of hyperopia in small eyes has frequently been attributed to an artifact of reflections used in measurements originating from the inner retinal surface, our approach allowed us to rule out this possibility by analyzing the spot pattern images and selecting spots from the deeper retinal layer whenever a dual spot pattern was observed. Furthermore, the majority of spot-pattern images captured from our guinea pig subjects and subsequently analyzed did not exhibit a dual spot pattern, which is commonly observed in mice.^25^

Although it is now well accepted that the growth of young eyes is actively regulated to reduce and/or eliminate neonatal refractive errors, a process known as emmetropization, the nature of the optical information used to decode the defocus experienced by the retina remains poorly understood.^26^^–^^27^ The measurement of the optical aberrations of the guinea pig eye represents an important step forward toward allowing the nature of the defocus stimuli and their effects on retinal image quality to be better understood for this increasingly popular mammalian model.

Comparison of the wave aberration contour plots derived from our eight guinea pigs suggests a degree of randomness with respect to inter-animal differences, based on the Zernike coefficients computed over a 4-mm pupil. However, it is also noteworthy that, after the exclusion of the data from the one animal with cataracts, the derived group averages of individual Zernike coefficients were mostly close to zero. Variations in aberrations also appear to be to some extent random for human eyes, albeit small in size. For example, compiled statistics from one study of 2560 human eyes show that, of all high-order aberration terms, only spherical aberration was found to be non-zero.^28^ The small positive spherical aberration reported in the aforementioned study was similar to our finding in the guinea pig eye (0.0481 ± 0.077). Although none of our data reached statistical significance, this may reflect the small number of guinea pig eyes included in the current study. Also consistent with findings for human eyes,^29^ the crystalline lens of the guinea pig exhibits significant negative spherical aberration,^7^^,^^30^ implying that the corneal contribution is positive spherical aberration, as reported here for spherical aberration overall.

Of the mammalian models used in myopia research today, rodent models have become increasingly popular, with mice and guinea pigs emerging as the two most common. As noted in the introduction, the visual acuity of guinea pigs is only slightly better than that of mice, despite their much larger eye size. These observations raise the question of how much of its poor performance can be attributed to differences in the optical quality of their eyes. As the guinea pig is used as a model to make predictions about human ocular development, we undertook relevant comparisons of guinea pig and human eyes. Specifically, we compared the RMS wavefront errors of different terms, after excluding second-order aberrations (defocus and astigmatism), that are most deleterious to vision but also correctable by standard optical means. For both guinea pig and human eyes, RMS errors decreased with increasing order, although they were consistently larger for the guinea pig eye. Nonetheless, RMS errors for the guinea pig eyes were only four times the estimates for the human eyes (Fig. 3). The relatively high optical quality of the guinea pig eye contrasts with their relatively poor visual acuity, which presumably has a neuroanatomical origin.

In relation to retinal image quality, pupil size plays an important role. Compared to both humans and also young chicks, guinea pigs have naturally large pupils under photopic conditions. This is reflected in our choice of a 4-mm pupil size for follow-up analyses that aimed to characterize the visual experience of guinea pigs under laboratory lighting conditions. In contrast, in the related laser ray tracing (LRT) study of the guinea pig eye, data analysis was limited to a 2-mm pupil size.^13^ Although, in that study, the reported average RMS error values of 0.10 and 0.18 µm, as measured by LRT and derived from an OCT-based simulation, respectively, are much smaller than our estimate of 0.475 µm for a 4-mm pupil, our estimate when scaled down to a 2-mm pupil is 0.167 µm, which falls within the same range (slightly higher than the LRT measurement but lower than the OCT simulation). However, given our finding that RMS errors increase rapidly with increasing pupil size for the guinea pig eye (see Fig. 6A), the use in optical modeling of pupil sizes that more closely represent those encountered in awake animals would seem advisable in the context of ocular growth regulation and myopia.

**Figure 7.**
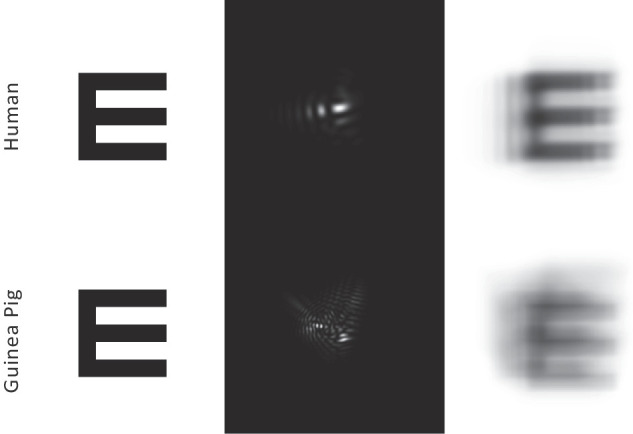
Five-arcmin letter E shown alongside PSFs and convolved images for a representative human eye (S09, *top row*) and guinea pig eye (#3, *bottom row*). Calculations used a 4-mm pupil size and 550-nm wavelength.

As one approach for comparing the optical quality of guinea pig and human eyes, we derived Strehl ratios for both species. Overall, the values representing guinea pig eyes (0.031–0.019) were much lower than those for human eyes (0.61–0.078). As another way of illustrating this difference in the optical quality of guinea pig and human eyes, we show in Figure 7 a 5-arcmin (20/20) letter E convolved with a PSF from representative human and guinea pig subjects. The difference in retinal image quality between them is quite apparent; the blur is greater, and the contrast is reduced for the guinea pig simulation, as is expected given the lower Strehl ratio and higher magnitude of high-order aberrations. Nonetheless, the 20/20 letter is still legible, even though it is far smaller than the visual acuity of the guinea pig, as measured behaviorally. As an aside, our choice of a standard letter E for these calculations reflects its wide use in human spatial resolution studies and thus familiarity within the vision research community. With the exception of celestial objects (e.g., sun, moon), real-world images vary widely in their spatial profiles and are often of arbitrary visual angles, making the results difficult to interpret.

As evidence of active emmetropization in young guinea pigs, they have been shown to compensate for defocus imposed artificially with lenses through adjustments to eye growth.^3^ What is the significance of the optical aberration and derived depth of focus data for the guinea pig eye reported here? Depth of focus is generally defined as the variation in defocus that can be tolerated by the eye without causing any notable change in sharpness of the retinal image. Here, we derived depth of focus values for both guinea pig and human eyes from Strehl ratios, which correlated reasonably well with perceived image quality, at least for humans.^31^ Using this approach and a 50% threshold, the depth of focus for the guinea pig eye was estimated to be 4 D; yet, very young 2- to 3-day-old guinea pigs have been shown to respond with ocular growth changes to as little as 2 D of imposed optical defocus.^3^ Therefore, these results suggest that the retina may respond to very small changes in contrast or use other optical cues, such as chromatic aberration, to decode and signal defocus experiences.

**Figure 8.**
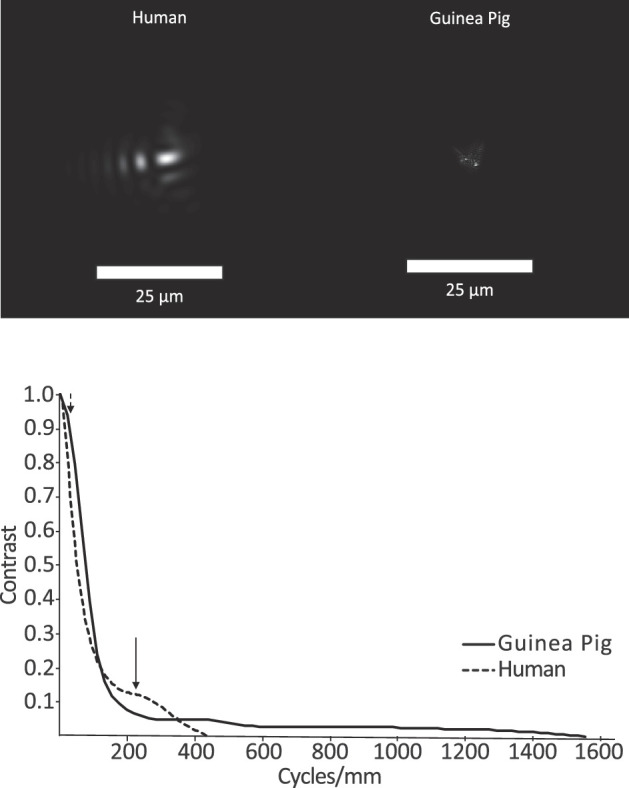
(A) PSFs of representative human eye (S009, *left*) and guinea pig eye (#3, *right*), shown in linear units. Although the guinea pig eye suffered from more high-order aberrations than the human eye, the physical size of its PSF was smaller, due to its shorter focal length. (**B**) The radial average MTFs plotted in cycles/mm for guinea pig (*solid line*) and human (*dashed line*) eyes. The peak cone sampling frequencies are indicated by the *arrowheads* (corresponding to the human fovea, 29 cycles/mm, and guinea pig visual streak, 215 cycles/mm). Calculations used a 4-mm pupil size and 550-nm wavelength.

Although the guinea pig eye is more aberrated than the human eye, as measured by RMS errors, the extent of the PSF on the retina is smaller, due to its shorter focal length or higher numerical aperture (Fig. 8A). To further understand how this difference in eye length might affect retinal image processing in guinea pig compared to human eyes, the radial average MTFs were replotted in cycles/mm (linear units) for both (Fig. 8B). The sampling resolutions of both guinea pig and human eyes were first estimated from reported retinal sampling density data. Retinal ganglion cell density maps have proven to be better predictors of spatial resolution than equivalent photoreceptor density maps across a wide range of species and so were used here for the guinea pig.^32^ Specifically, a maximum ganglion cell density of 2272 cells/mm^2^, corresponding to the visual streak of the guinea pig,^33^ was used. Making the simplistic assumption that the ganglion cells are optimized to subserve spatial vision (i.e., receptive fields evenly tiling the retinal surface with hexagonal packing), the upper bound on the sampling resolution would be 26 cycles/mm as given by the formula:

$$\;Sampling\;resolution=\;\frac{1}{2}\;\sqrt{\frac{2}{\sqrt{3}}\;\left(sampling\;density\right)}$$

The latter estimate translates to a visual acuity of 2.4 cpd (1 degree of visual angle = 82 µm across the retina in a guinea pig), which correlates well with behavioral visual acuity estimates. Equivalent calculations for the human eye used an average foveal cone density of 163,000 cones/mm^2^,^12^ which translates to a sampling resolution of 215 cycles/mm and 62.5 cpd. Therefore, although optics limit human spatial vision, retinal ganglion cell density appears to limit the visual acuity of the guinea pig.

When plotted in linear units (cycles/mm), the high-frequency cutoff of the MTF for the guinea pig eye was greater than for the human eye. These transformed data also reveal a slight contrast advantage for the guinea pig eye over the human eye for low spatial frequencies where the guinea pig's peak sensitivity lies. The significance of the latter result for eye growth regulation remains unknown. Nonetheless, these different analyses (spatial vs. angular) warrant further consideration in the context of emmetropization and the cues that guide it.

This study has several important limitations. First, this study used just one strain of pigmented guinea pig, which is of New Zealand origin. Reports of differences in sensitivity to myopia-inducing stimuli among pigmented guinea pig strains and significant refractive error-related developmental differences between pigmented and albino guinea pigs raise the question of the generalizability of the results reported here. Nonetheless, we did see close correspondence between the average RMS error values for our guinea pigs and a 2-mm pupil diameter and that reported in the only other relevant study involving guinea pigs. Second, the data reported here were collected following cycloplegia, to eliminate potential accommodative influences. In human eyes, spherical aberration becomes more negative as accommodation increases, but the average change for the other Zernike terms is minimal.^15^ Given that guinea pigs are known to accommodate,^7^ it would be of interest to know whether their accommodation similarly affects their optical aberrations, especially given that the relatively closed environment in which they are raised in studies of eye growth regulation necessarily lends itself to regular accommodative activity. Finally, this was a cross-sectional study on adolescent guinea pigs, and it would be of interest to see if longitudinal changes occur, particularly when guinea pigs are undergoing emmetropization.

## Conclusions

Although visual acuity is much poorer in the guinea pig compared to the human eye, high-order aberrations are not major sources of optical quality degradation. Importantly, the optical quality data reported here for the guinea pig were based on their natural pupil size and represent an important resource for future studies on optical defocus regulation of eye growth using this model. The comparative data derived from spatial versus angular analyses of optical quality offer an additional new perspective on how optical aberrations impact vision.
